# Attitudes of Italian Psychiatrists Toward the Evaluation of Physical Comorbidities and Sexual Dysfunction in Patients With Schizophrenia. Implications for Clinical Practice

**DOI:** 10.3389/fpsyt.2019.00842

**Published:** 2019-11-20

**Authors:** Palmiero Monteleone, Mario Amore, Aderville Cabassi, Massimo Clerici, Andrea Fagiolini, Paolo Girardi, Emmanuele A. Jannini, Giuseppe Maina, Alessandro Rossi, Antonio Vita, Alberto Siracusano

**Affiliations:** ^1^Department of Medicine, Surgery and Dentistry, “Scuola Medica Salernitana”, University of Salerno, Salerno, Italy; ^2^Section of Psychiatry, Department of Neuroscience, Ophthalmology, Genetics, and Infant-Maternal Science, IRCCS Ospedale Policlinico San Martino, University of Genoa, Genoa, Italy; ^3^Department of Clinical and Experimental Medicine, Centro Studio dell'Ipertensione Arteriosa e delle Malattie Cardiorenali, Clinica e Terapia Medica, University of Parma, Parma, Italy; ^4^Department of Medicine and Surgery, University of Milano Bicocca, Milan, Italy; ^5^Department of Molecular Medicine, University of Siena, Siena, Italy; ^6^Department NESMOS, Sapienza University of Rome, Rome, Italy; ^7^Department of Systems Medicine, University of Rome Tor Vergata, Rome, Italy; ^8^Rita Levi Montalcini Department of Neuroscienze, San Luigi Gonzaga University Hospital, University of Turin, Turin, Italy; ^9^Department of Mental Health, University of L'Aquila, L'Aquila, Italy; ^10^Department of Clinical and Experimental Sciences, University of Brescia, Brescia, Italy; ^11^Department of Medicine Systems, University of Rome Tor Vergata, Rome, Italy

**Keywords:** schizophrenia, physical comorbidities, sexual dysfunction, cardiometabolic risk, antipsychotic, prolactin, psychometric test, survey

## Abstract

Treatment guidelines for patients with schizophrenia recommend evaluating their risk of physical comorbidities, especially since these patients are known to have decreased life expectancy due to comorbidities. Therefore, to the authors’ knowledge, this is the first national survey conducted to investigate how Italian psychiatrists deal with the risk of physical comorbidities and sexual dysfunction in patients with schizophrenia. A sample of 750 psychiatrists completed an *ad hoc* online survey investigating their decision making about performing blood tests, clinical and instrumental examinations, and scheduling follow-up appointments in relation to the different phases of the illness and possible pharmacological side effects. Compared to patients in therapeutic continuation, those diagnosed for the first time and those who received a therapeutic change were visited more frequently (every 15 to 17 days *vs.* every 40 days, respectively), and were more regularly prescribed blood tests and instrumental examinations (every 4.2 to 4.4 months *vs.* every 9 months, respectively). There was a high interest in the surveillance of cardiometabolic risk. In 54% of patients, prolactin testing was not requested before starting an antipsychotic. In terms of specialist referrals, only 5% of surveyed psychiatrists “never” sought for additional counseling. There was little attention given to sexual functioning assessment based on the survey results about patients' daily life and with regard to deciding to prescribe additional examinations. In fact, only up to 3% of psychiatrists reported assessing sexual functioning using specific psychometric tests. In summary, Italian psychiatrists describes themselves as careful healthcare providers for the physical illnesses of patients with schizophrenia but with several shortcomings. For instance, clinical attention toward patients’ sexual and reproductive healthcare needs remains a challenge. Psychiatrists should take the lead for the integrated education, assessment, and care of medical needs of their patients with mental illness. Based on the results of this survey, the authors also believe that a future challenge for the management of patients with mental illness will be the classification of patients based on their risk of comorbidities, to help ensure optimal healthcare provision for those at greater risk of other illnesses.

## Introduction

In persons with schizophrenia, the high prevalence of medical comorbidities and resulting mortality is an alarming and worldwide phenomenon with negative short- and long-term health effects (1). Compared to the general population, life expectancy may be shortened by 10 to 20 years due to the increased risk of physical comorbidities was reported (2). The high prevalence of comorbidities has been conceptualized as the result of several factors such as side effects of pharmacological treatments and the adoption of unhealthy lifestyle behaviors characterized by physical inactivity, poor quality of food intake, smoking habits, and reduced social functioning (3). However, it is also widely known that physical well-being of people with schizophrenia has been historically ignored, including data for monitoring basic parameters like blood pressure and body weight (4).

The inadequate monitoring and treatment of metabolic abnormalities was previously documented by the CATIE study (5). A large Danish population-based study from 2000 to 2012 revealed that for people with a first-time schizophrenia diagnosis, the highest reported rate of metabolic monitoring was 71%, with more than a half of patients having an abnormal lipid profile and more than 10% with blood glucose imbalance (6). There is growing evidence that persons with schizophrenia continue to be victims of healthcare disparities due to their decreased healthcare access and use, and healthcare professionals’ insufficient consideration of the poor physical health outcomes of the physical illnesses and lifestyle choices in this group of people (7). Moreover, addressing sexual dysfunction in persons with psychotic disorders remains an unmet need but a key health determinant (8). For instance, surveillance and pharmacological care of sexual dysfunction were reported to be lacking in patients with mental disorders (9).

These shortcomings in healthcare access and provision may contribute to life-threatening events that need to be addressed. Thus, improvement of physical comorbidities in people with schizophrenia is now considered to be a necessary management goal and has been included in treatment guidelines (10). Therefore, for the first time, we conducted a national survey investigating how Italian psychiatrists who treat persons with schizophrenia deal with their patients’ physical comorbidities and sexual dysfunctions. The primary goal of the survey was to investigate psychiatrists’ decision making about performing blood tests and clinical and instrumental examinations, and scheduling follow-up appointments with respect to the different phases of their patients’ illness and possible pharmacological side effects.

## Materials and Methods

### Study Participants

Participants were 750 Italian psychiatrists working in different clinical settings, mainly public mental health services (see below **Table 1**). Participation was voluntary. Study participants were enrolled by distributing a brochure containing a brief summary of the research proposal and the link to the online survey. The flyer was allocated in conferences, outpatient and inpatients clinics. Once logged, the psychiatrists were authorized to withdraw a portion of or their entire response to the survey if they decided to pull out from the study; consequently, any data or recordings obtained from these participants were destroyed and were not allowed to be used in any resulting publication. Psychiatrists were also informed that the answers to the survey were confidential and anonymous. They were guaranteed that none of the information provided would be used for direct marketing or other non-research activities. Given the design of recruitment, psychiatrists were free to decline their participation to the study without addressing it. Accordingly, the response rate to the survey was not available. Ethics approval was not required as per local legislation and national guidelines.

**Table 1:** Demographics of study participants.

Percentage		
Sex		
Male	54%	(N = 407)
Female	46%	(N = 343)
Age (years)		
<40	25%	(N = 188)
41–50	23%	(N = 174)
51–60	31%	(N = 236)
>60	20%	(N = 152)
Years of activity		
<10	26%	(N = 192)
11–20	25%	(N = 191)
21–30	31%	(N = 231)
>30	18%	(N = 136)
Region of provenance		
Northern Italy	44%	(N = 328)
Central Italy	23%	(N = 174)
Southern Italy and Islands	33%	(N = 248)
Practice setting		
Outpatient clinic	61%	(N = 455)
Inpatient setting	28%	(N = 208)
Residential setting	6%	(N = 44)
Private practice	6%	(N = 43)

### Survey

Data were collected *via* computer-assisted telephone interviewing (C.A.T.I.) and computer-assisted web interviewing (C.A.W.I.) of a random group of 750 psychiatrists, from February 2018 to February 2019. The authors representing a scientific committee of national experts with a special interest in the comorbidities of people with schizophrenia drafted an *ad hoc* 15-min questionnaire based on published guidelines for writing surveys (11, 12). The complete English translation of the survey may be referred to in the **Supplementary Materials**, and includes questions about:

- The characteristics of the psychiatrists (gender, age, years of working experience, working in inpatients *vs.* an outpatient setting, Italian region of provenance)
- The characteristics of the psychiatrists’ activity (number of patients seen per months, percentage of patients with schizophrenia)
- The characteristics of patients affected by schizophrenia (phase of illness, number of patients with known *vs.* first-time diagnosis of schizophrenia)
- Percentage of patients for whom psychiatrists decided for therapy continuation against patients in which they preferred changing therapy, in terms of adding or removing a drug or changing its dosage. Factors influencing the decision to change the treatment (efficacy, side effects, physical comorbidity, others)
- Information about pharmacological side effects that are declared by patients (type of side effects, side effects reported as more disturbing)
- Frequency of clinical assessments, prescriptions of blood tests, instrumental examination, and clinical referral
- How often the psychiatrists ask the patient about physical comorbidity indicators and pharmacological side effects (sleep disorders, tremors, body weight changes, sexual dysfunctions, waist circumference, gynecomastia, family history of cardiovascular disease, musculoskeletal disorders, urinary dysfunctions, respiratory tract diseases)
- How often the psychiatrists ask the patient about unhealthy lifestyle behaviors (dietary patterns, physical activity, substance abuse, behavioral addiction such as sex, computers, and gaming, smoking habits, alcohol abuse, misuse of psychotropic-prescribed medications, private relationships, sexual activities, sexual orientation, functioning in relation to self-care)

### Analysis

Data collected from the web-based survey tool were analyzed using the SAS (Statistical Analysis System) for data control. The statistical tables were constructed by NIPO Diana System (NIPO Software, a Kantar Company).

The chi-squared (χ^2^) test with 95% confidence interval was used for statistical significance calculation between percentages. The Student's *t*-test with 95% confidence interval was used for statistical significance calculation between means.

## Results

### Characteristics of the Participant Psychiatrists

Sample characteristics are presented in **Table 1**. Of the 750 psychiatrists included in this non-interventional study, 54% were men and 46% were women. The mean age was 50 (± 11.26) years (range: 25–76), the mean years of working experience was 20 (± 11.36) years (range: 1–50). The provenance of participants was equally spread across Italy. The majority of participants worked in an outpatient's public setting.

### The Characteristics of the Psychiatrists’ Activity (Total Sample)

Each psychiatrist visited a mean number of 96 (± 71.2) patients per month (range: 10–600), of whom 28% had schizophrenia, 28% had bipolar disorder, and 44% had other mental disorders.

### The Characteristics of Patients Affected by Schizophrenia

Three out of 10 patients visited by clinicians suffered from schizophrenia (min–max: 1–280). With regard to the course of illness, approximately two out of three patients (min–max: 1–100) were in a stable phase and one out of three were in an acute exacerbation phase of illness (68% as against 32%). Psychiatrists reported that of a mean of 100 patients visited monthly, approximately 14% (min–max: 1–100) received a diagnosis of schizophrenia for the first time, 30% (min–max: 1–100) underwent treatment changes, and 55% (min–max: 1–100) received treatment confirmation.

### The Factors Influencing Psychiatrists Deciding to Change the Therapy

Of all the respondents, about 34% of psychiatrists reported that they changed patient’s therapy due to side effects, 19% due to physical comorbidities, and 47% due to treatment failure or inadequate efficacy.

### Information About Pharmacological Side Effects Reported by Patients

**Figure 1:**
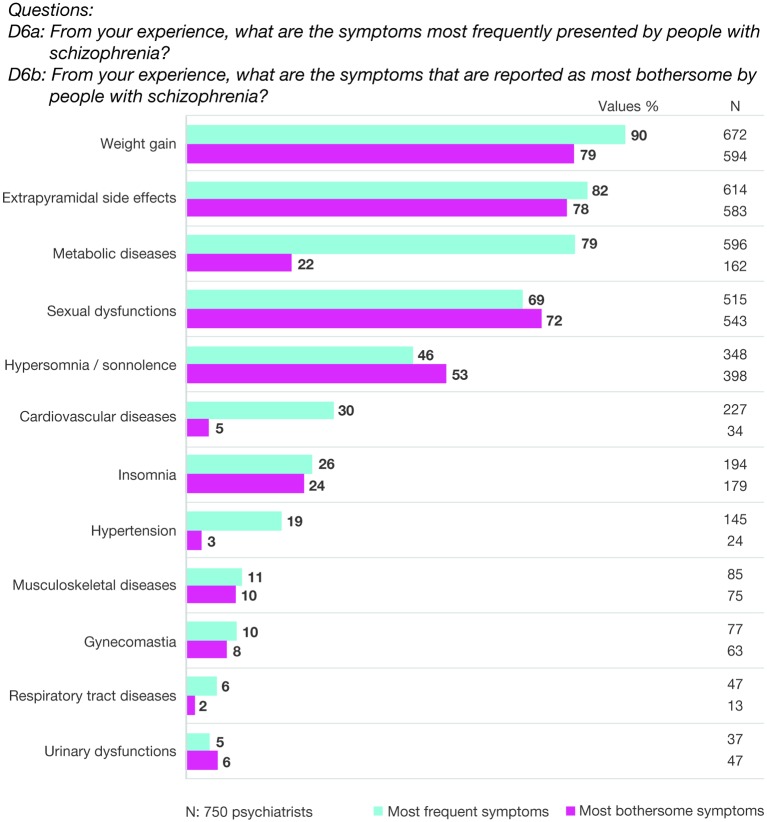
Most frequent and bothersome problems for people with schizophrenia, as reported by psychiatrists.

According to the psychiatrists, weight gain, extrapyramidal side effects, and sexual dysfunction were the most reported and most bothersome symptoms for patients with schizophrenia. Metabolic problems were also frequent (79%) but fewer patients reported it as bothersome (22%). Fewer patients also reported cardiovascular problems and arterial hypertension as bothersome (i.e., 5 and 3%, respectively) (**Figure 1**).

### Frequency of Clinical Assessments, Prescriptions of Blood Tests, Instrumental Examinations, and Clinical Referral

**Figure 2:**
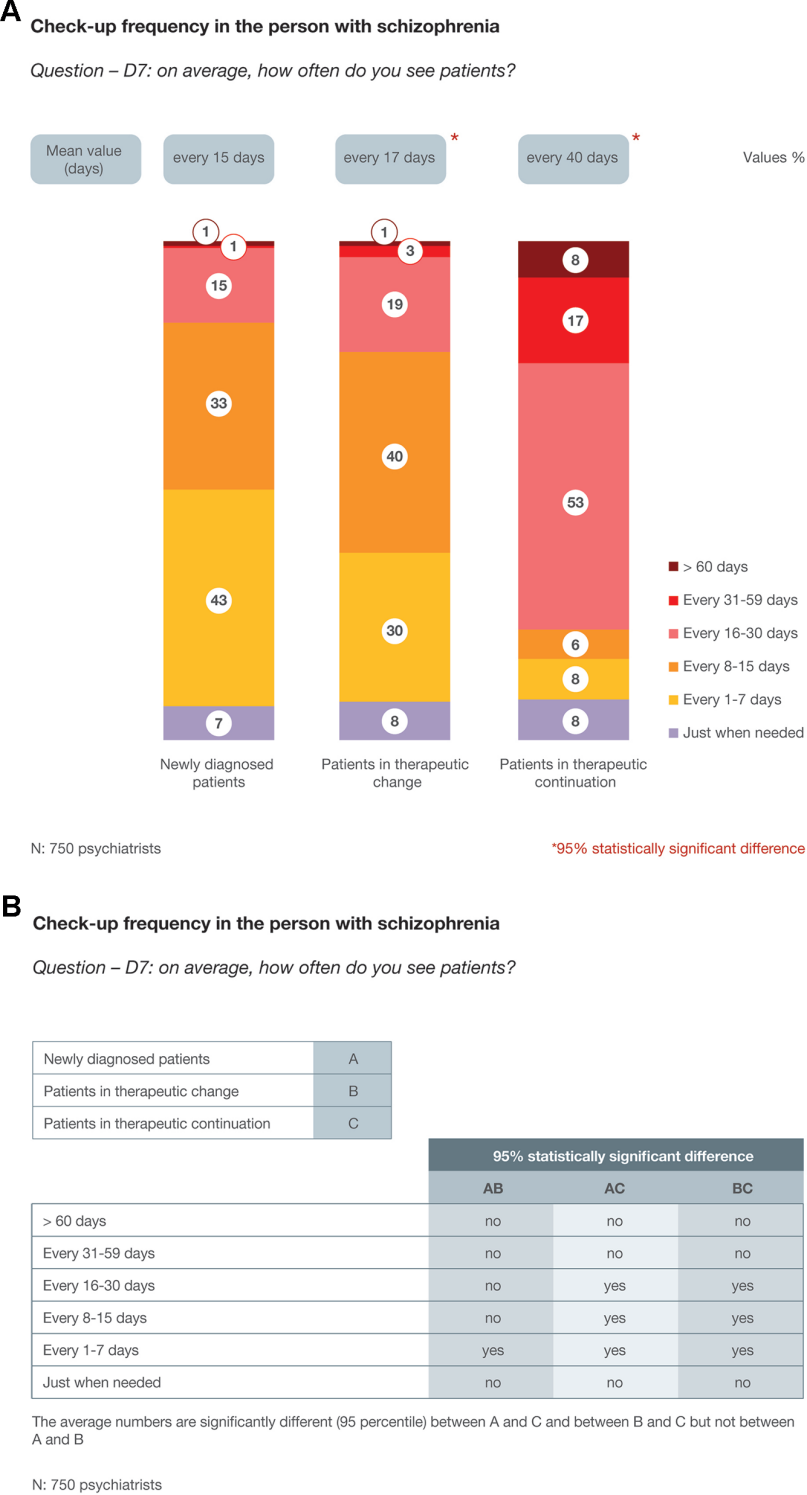
**(A)** Check-up frequency in patients with schizophrenia. **(B)** Check-up frequency in patients with schizophrenia: the differences between groups (χ^2^ test).

As shown in **Figure 2A**, patients who received a diagnosis for the first time and patients who have changed therapy were visited more frequently compared to patients in therapeutic continuation (every 15 to 17 days *vs.* every 40 days, respectively). A χ^2^ test was performed to assess the differences between the three groups, as shown in **Figure 2B**.

In the three groups of patients the examinations more frequently required were electrocardiogram and measurements of blood cholesterol, triglycerides, glucose, and liver enzymes. Prolactin level evaluation was requested more often when the psychiatrist decided to change the therapy (**Table 2**, question—D9: In detail, what tests do you require for the three different types of patients?).

**Table 2:** Required examinations for the different types of patients with schizophrenia.

Question: In detail, what tests do you require for the three different types of patients?			
	Patients being diagnosed for the first time^1^	Patients in therapeutic change^2^	Patients in therapeutic continuation^3^
Electrocardiogram	96*	89*	86*
Cholesterol blood level	89*	81*	86*
Fasting blood sugar level	88*	82*	79*
Triglycerides blood levels	88*	80*	81*
Liver enzymes levels	86*	80*	79*
Blood pressure	80*	72*	68*
Blood tests of renal function	77*	71*	69*
Serum electrolyte levels	75*	67*	64*
Thyroid hormone levels	69*	53*	45*
Prolactin levels	56*	69*	59*
Illicit drug metabolites	43	22	14
HIV-hepatitis testing	27	13	9
Prostate-specific antigen test	9	6	8
Sex hormones	6	5	5
Mammography	3	2	4
PAP test	3	2	3
Psychometric tests for sexual functioning	3	2	2
Fecal occult blood test	2	2	1

^1^Percentage based on a total number of 718 psychiatrists. ^2^Percentage based on a total number of 712 psychiatrists. ^3^Percentage based on a total number of 711 psychiatrists. *95% statistically significant difference.

**Figure 3:**
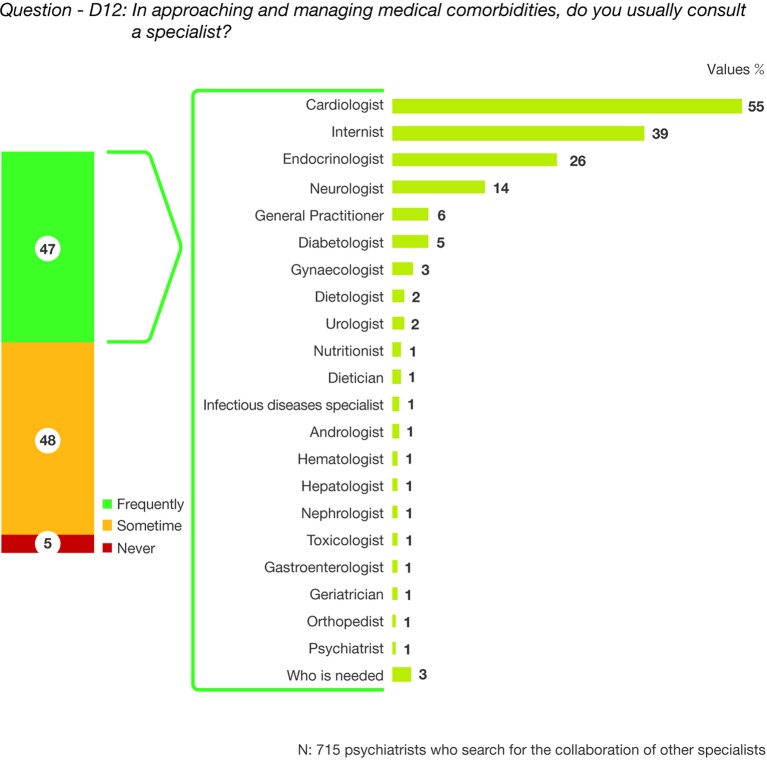
Question—D12: In approaching and managing medical comorbidities, do you usually consult a specialist?

The analysis of comorbidity management in collaboration with other specialists showed that 47% of responders “frequently” asked for a specialist referral. Cardiologists, internal medicine specialists, and endocrinologists were the most frequently involved specialists. (**Figure 3**, question—D12: In approaching and managing medical comorbidities, do you usually consult a specialist?).

### Frequency of Assessment of Troublesome Issues in the Patients With Schizophrenia

**Figure 4:**
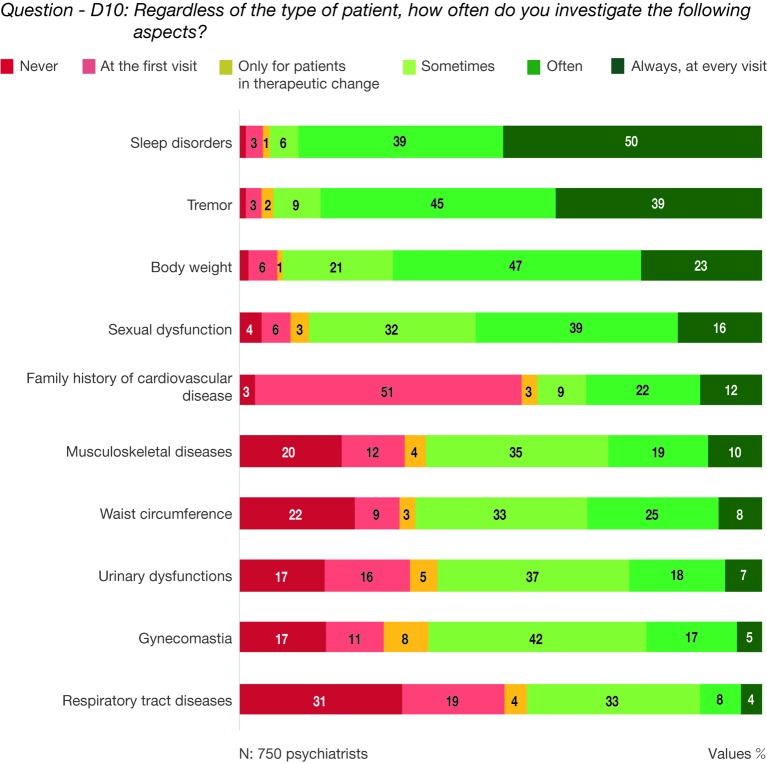
Question—D10: Regardless of the type of patient, how often do you investigate the following aspects?

Sleep disturbances and tremors were the most investigated aspects. Body weight and sexual dysfunctions were also assumed as very important by the specialists. The family history of cardiovascular disease was mainly investigated at the first visit (**Figure 4**, question—D10: Regardless of the type of patient, how often do you investigate the following aspects?).

### Frequency of the Analysis of Aspects Related to the Patient's Everyday Life

**Figure 5:**
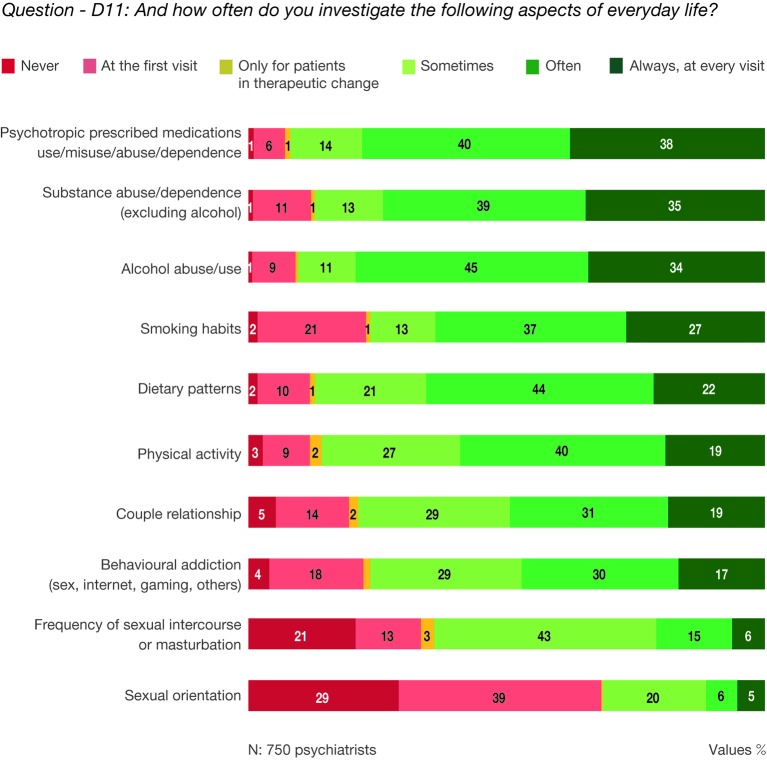
Question—D11: And how often do you investigate the following aspects of everyday life?

Use of psychotropic prescribed drugs, substances, and alcohol, including abuse, misuse, and/or dependence, were the most commonly investigated aspects of patient's daily life. Sexual function was the most highly neglected aspect by clinicians (**Figure 5**, question—D11: And how often do you investigate the following aspects of everyday life?).

## Discussion

In persons with schizophrenia, premature mortality is primarily due to preventable physical diseases. Hence, the aim of this survey was to understand the current attitudes of Italian psychiatrists regarding this threatened population. The sample of our study included clinicians working in different clinical settings, including outpatient and inpatient care, from across Italy, to provide a closer picture of their real-life practice. We aimed to explore if the psychiatrists were adequate in inquiring about comorbidities and lifestyle habits, in prescribing blood tests, instrumental examinations, and psychometric questionnaires. We also investigated if there was an intent of minimizing the risk of comorbidities from the beginning of the therapeutic process. Furthermore, we examined the frequency pattern of patient assessments and laboratory examinations throughout the various phases of the patients' illness, specifically in first-time diagnosis patients, patients in therapeutic change, and patients in treatment continuation.

We found a monthly fairly high-incidence of first time diagnosis of schizophrenia (14%) and a high rate of pharmacological switchers (30%). This result may be due to the psychiatrists’ clinical setting of provenience. In fact, the majority of participants worked in acute inpatients setting and outpatients units that were characterized by a high rate of check-up.

Patients diagnosed for the first time with schizophrenia and those who were received a therapeutic change were visited more frequently *vs.* patients in therapeutic continuation (every 15–17 days *vs.* every 40 days, respectively). Similarly, blood tests and instrumental examinations were more regularly prescribed for those diagnosed for the first time and those who were received a therapeutic change *vs.* those in therapeutic continuation (every 4.2–4.4 months *vs.* every 9 months, respectively). Regarding the kind of examinations required, we observed that, in general, psychiatrists tend to prescribe more examinations for the newly diagnosed patients *vs.* the other patient groups. Meanwhile, blood prolactin value was more frequently requested for patients in treatment change *vs.* those in the newly diagnosed group. Hence, it appears that more than half of patients (54%) are not stratified according to basal prolactin levels before starting an antipsychotic treatment. This is an important finding because treatment with first- and second-generation antipsychotics is often associated with hyperprolactinemia, warranting the need to obtain baseline prolactin measurements and constant monitoring to guide follow-up therapy decisions and shifting strategies (13).

On the other hand, Italian psychiatrists showed high interest in screening for cardiovascular disease risk, including obtaining an electrocardiogram and measuring patients’ total lipid profile and blood pressure. In particular, the close monitoring of the corrected QT interval was in line with the recommendation of the Italian Medicines Agency (AIFA) that is the national authority responsible for drugs regulation in Italy. It is worth noting that in people with schizophrenia, the prevalence of cardiovascular disease was found to be 2.5 times higher than the general population (14). Moreover, while the last four decades has shown a global reduction in cardiovascular mortality (15), this has not been the case for patients with severe mental disorders (16).

The surveyed psychiatrists also often examined patients’ metabolic profile in terms of glycemia, triglyceridemia, cholesterolemia, and body weight. Weight gain, in particular, was conceived as the most common and bothersome problem for patients. Previous data of patients survey on subjective quality of life showed that the diagnosis of schizophrenia was correlated with low physical functioning (17). Moreover, patients with metabolic syndrome and schizophrenia perceived a higher number of care needs, lower global functioning score, and poor adherence to treatment (18–20). There is general acquiescence that treatment with second-generation antipsychotics may cause weight gain in patients with schizophrenia (21); however, weight gain in general is multifactorial and is affected by socioeconomic disadvantages, unhealthy lifestyle, and premorbid genetic vulnerabilities.

Schizophrenia *per se* may be an independent risk factor for metabolic disorders and obesity (22). In fact, a “developmental” type of obesity has been proposed in the 1950s as a defense against mental illness (23). Accordingly, turning to overeating and obesity may be conceived as protections from premorbid threatening sensations and feeling that are characteristic of the schizophrenic development. To date, PubMed has indexed over 2,000 manuscripts on the topic of weight gain and schizophrenia. For example, available literature proposes that insulin resistance occurs before obesity development and the use of antipsychotic medications, although these likely play a complicating key role later in the progression of schizophrenia (24).

In summary, our results confirm that surveyed Italian psychiatrists recognize the importance of effective monitoring of patients’ cardiometabolic risk. Data was particularly encouraging compared to previous epidemiological studies, which reported a mean of 8 to 30% testing rates in person with serious mental illness who started treatment with a new antipsychotic (25, 26). However, while the surveyed Italian psychiatrists reported that weight gain was a frequent presentation in patients with schizophrenia (79%), on the other hand they believed that patients underestimated the detrimental impact of metabolic problems, with only 22% of cases reporting weight gain as bothersome. The apparent unawareness of patients regarding the important consequences of metabolic abnormalities may serve as a catalyst for mental health professionals to educate patients, their family, and caregivers, and provide *ad hoc* psycho-education interventions (27).

The analysis of the collaboration with other specialists showed that almost half of responders “frequently” (47%) and “sometimes” (48%) asked for a specialist referral, with only 5% “never” seeking additional consult. The most commonly consulted specialists were cardiologists, internists, and endocrinologists. Only 1% of the surveyed psychiatrists reported consultations with nutritionists or dieticians. Because available data documented that individuals with severe mental disorders are often victims of suboptimal healthcare systems (28, 29), our results about the psychiatrists’ “frequently” turning to medical specialists was reasonable. The factors contributing the inequitable access to physical care and treatment are multiple, including difficulties with interactions between healthcare providers, separation of responsibilities and disorganized care, and patient's barriers to recognizing the need for specialist intervention. Many people with severe mental illness also experience discrimination due to their diagnosis, including from general practitioners or in the general healthcare context (30). Moreover, psychiatrists may feel uncomfortable addressing and managing comorbidities. Nevertheless, psychiatrists are crucial in the overall health of persons with schizophrenia because they are often the only health provider these patients will see (31). In this perspective, it is cogent for mental health providers to become competent in managing their patients’ physical health through proper training programs. Taking these into consideration, psychiatrists should ideally take the lead for the integrated education, assessment, and care of medical needs of their patients with mental illness.

Care for persons with severe mental illness is complex and highly interdisciplinary. It involves a variety of health professionals, including cardiologist, internist, endocrinologists, gynecologists, urologists, dieticians, and toxicologists. Based on the results of this survey, the authors believe that a future challenge for the management of patients with mental illness will be the classification of patients based on their risk of comorbidities, to help ensure optimal healthcare provision for those at greater risk of other illnesses. Endorsement from Italian medical societies and specialist associations may be beneficial in producing such guidelines and recommendations.

In this survey Italian psychiatrists describes themselves as careful healthcare providers for the physical illnesses of patients with schizophrenia, but with several substantial shortcomings. For instance, surveyed psychiatrists were mainly focused on substance and alcohol use disorders. Comorbid substance use disorders were associated with poorer clinical outcome and it might influence tolerability, efficacy, choice, and formulation of antipsychotics (32, 33), supporting the need to assess these dual disorders in patients with mental illness. However, the survey also revealed that psychiatrists did not put the same emphasis on the other important aspects of their patients’ daily life, including smoking and dietary habits, physical activity, behavioral addictions, and partner relationships.

In particular, it is important to highlight that sexual function was not frequently assessed by the surveyed psychiatrists when asked about their patients' daily life and regarding their decision of prescribing additional examinations. Up to 3% of prescribers reported assessing sexual functioning using specific psychometric tests, although a number of specific and well-validated tools are available and used outside the psychiatric community such as the International Index of Erectile Function (IIEF) and the Female Sexual Function Index (FSFI) that are self-report measures of sexual function (34, 35). This reluctance to monitor sexual activities has been previously reported (36) and, unfortunately, does not appear to be a priority for Italian specialists. Many psychiatrists and doctors in general are not trained to address sexual dysfunction and may thus feel embarrassed and uncomfortable addressing this topic (37). According to a report, low awareness of and attention to sexual dysfunction may have significant negative impacts on patients (38). However, sexual dysfunction assessment remains a challenge. The psychometric tools for assessment of sexual dysfunction are simple, easy to perform, and inexpensive, and consequently should be implemented in the healthcare systems (35). Moreover, health workers other than specialists may routinely administer these simple tests (39). Psychometric instruments addressing male and female sexual health can also indirectly but efficaciously reveal important information about the presence of non-communicable chronic diseases and lifestyle disorders (40–42). Therefore, psychiatrists need to understand that investigating these areas not only responds to an unmet need of patients, but may also help in uncovering other critical physical comorbidities. In fact, sexual dysfunctions can be secondary to the disease itself or to comorbid physical disorders, or it may be an adverse effect of psychotropic drugs (43, 44). Importantly, the little attention given to the sexual sphere may have negative repercussions on patients' adherence and satisfaction with treatment, quality of life, and partner relationships (45). Although it was reported that people with stable schizophrenia were prone to rate their quality of life higher than healthy controls or clinicians would (46), high percentage of sexual dysfunction has been documented in the majority of patients with schizophrenia with negative impact on social adjustment and quality of life (47). Sexual functioning is also an important component of the bio-psycho-social model of health, and is recognized also by the World Health Organization as a part of general health (36). Thus, sexual health should be considered as part and parcel of modern psychiatry to provide a more comprehensive and holistic care for patients with mental health disorders (40, 48).

### Limitations

Survey results were based on psychiatrists’ responses to the survey and is thus limited by the lack of objective data to quantify the examinations requested by clinicians. The survey responses are also subject to several biases, such as “wishful thinking” answers. For instance, despite the anonymity of the survey, some psychiatrists may still claim to perform several laboratory tests to show that they follow current treatment guidelines. These biases may be overcome in future studies by including patients’ families, who may provide more accurate information about patients’ lifestyle habits that can be addressed to improve their quality of life (49). Direct access to patient's medical records may also help narrow the gap between the information provided by individual specialists and the objectifiable clinical reality. In addition, a more accurate report of the background of the psychiatrists, an investigation of the clinical activities and the characteristics of study centers may be useful.

Nevertheless, this survey provides a preliminary description of the attitudes and opinions of Italian psychiatrists that may influence clinical practice.

## Conclusions

In patients with schizophrenia, comorbidities and unhealthy lifestyle practices are associated with significant adverse physical and psychiatric outcomes (50). Therefore, routine clinical and laboratory monitoring are critical components of a comprehensive and integrated care program for this population.

This survey describes the quality of Italian psychiatrists’ care for patients with schizophrenia in terms of assessing comorbidities, frequency of medical assessments and use of available laboratory tests, frequency of clinical referrals, and use of psychometric tools for sexual dysfunction assessment. Mental healthcare providers should be responsible for the coordination of a multidisciplinary assessment and therapy, involving other medical specialists as needed. A successful strategy could be creating flow charts for integrated care pathways in patients with comorbidities or with high risk for comorbidities. Great attention must still be given to the patient's quality of life, especially concerning the sexual function area, with the aim of improving subjective well-being of people with severe mental illness (51, 52).

## Data Availability Statement

All datasets generated for this study are included in the article/**Supplementary Material**.

## Ethics Statement

Ethics approval was not required as per local legislation and national guidelines.

## Author Contributions

All authors contributed to the design of the study, analyzed and interpreted the results, revised the manuscript in each section, and approved the final version.

## Conflict of Interest

The authors declare that the research was conducted in the absence of any commercial or financial relationships that could be construed as a potential conflict of interest.
