# Supplementary material for: Attitudes of Italian Psychiatrists Toward the Evaluation of Physical Comorbidities and Sexual Dysfunction in Patients With Schizophrenia. Implications for Clinical Practice
Source: Front Psychiatry. 2019 Nov 20;10:842. doi: 10.3389/fpsyt.2019.00842 (PMC6879649; doi:10.3389/fpsyt.2019.00842)
Supplement: Supplementary file 1 [file Table_1.docx]

S.1 Do you work as a psychiatrist?

1. Yes
2. No 🡪 *give thanks and end the survey*

S. 2. Which one is your PREVALENT mental health treatment settings?

Outpatient clinic

Inpatient setting

Residential setting

Private practice

QUESTIONNAIRE

1. With regard to your activity as a psychiatrist, how many patients do you visit per month irrespective of the type of psychiatric disorder? Give the number of patients, not the number of checkups.

*Nella Sua attività di psichiatra, quanti pazienti visita in un mese medio, per qualsiasi tipo di patologia? Nel rispondere, consideri i pazienti una sola volta, non il numero di visite effettuate.*

Total number of patients ____________ per month

1. In particular, how many patients suffering from schizophrenia, bipolar disorder, and other mental disorders do you see per month?

*In particolare, di questi pazienti che Lei vede in un mese medio quanti sono affetti da Schizofrenia, quanti da Disturbo Bipolare e quanti da altre patologie?*

1. Schizophrenia ______number of patients

2. Bipolar disorder ______number of patients

3. Others ______number of patients

**Total = number of patients given in D1**

**Hereafter, please be focused on patients affected by SCHIZOPHRENIA**

1. Out of a mean of 100 patients suffering from schizophrenia that are seen every month for a check up, what is the percentage of those in stable phase and those in an acute exacerbation phase of illness?

1. Patients in stable phase ______% of patients

2. Patients in acute exacerbation phase ______% of patients

**Total = 100%**

1. Out of a mean of 100 patients visited or seen monthly, what is the percentage of those diagnosed with schizophrenia for the first time, those who underwent treatment changes, and those who received treatment confirmation?

1. Patients newly diagnosed ______% of patients

2. Patients in therapeutic change ______% of patients

3. Patients in therapeutic continuation ______% of patients

**Total = 100%**

1. Out of a mean of 100 patients visited or seen every month, what is percentage of those who underwent therapeutic changes due to side effects, physical comorbidities, or partial/absent clinical response?
2. % therapeutic change due to side effects ______% of patients
3. % therapeutic change due to comorbidities ______% of patients
4. % therapeutic change due to partial/absent clinical response ______% of patients
5. From your experience, what are the symptoms **MOST FREQUENTLY** presented by people with schizophrenia? **(multiple answers may be possible)**

|  | **Most frequent** symptoms reported by the person with schizophrenia |
| --- | --- |

| Weight gain |  |
| --- | --- |
| Metabolic diseases |  |
| Cardiovascular diseases |  |
| Hypertension |  |
| Sexual dysfunctions |  |
| Extrapyramidal side effects (parkinsonism, tremor, rigidity, dyskinesia, akathisia, dystonia) |  |
| Insomnia |  |
| Hypersomnia/somnolence |  |
| Gynecomastia |  |
| Respiratory tract diseases |  |
| Urinary dysfunctions |  |
| Musculoskeletal diseases |  |
| Others *(specify) __________________________* |  |

1. From your experience, what are the symptoms **MOST BOTHERSOME** presented by people with schizophrenia? **(multiple answers may be possible)**

|  | **Most bothersome** symptoms reported by the person with schizophrenia |
| --- | --- |

| Weight gain |  |
| --- | --- |
| Metabolic diseases |  |
| Cardiovascular diseases |  |
| Hypertension |  |
| Sexual dysfunctions |  |
| Extrapyramidal side effects (parkinsonism, tremor, rigidity, dyskinesia, akathisia, dystonia) |  |
| Insomnia |  |
| Hypersomnia/somnolence |  |
| Gynecomastia |  |
| Respiratory tract diseases |  |
| Urinary dysfunctions |  |
| Musculoskeletal diseases |  |
| Others *(specify) __________________________* |  |

1. **With regard to your patients affected by schizophrenia, on average, how often do you see each patient?**

…NEWLY DIAGNOSED PATIENTS: every |__|__| days or every |__|__| months

□ just when needed

…PATIENTS IN THERAPEUTIC CHANGE: every |__|__| days or every |__|__| months

□ just when needed

…PATIENTS IN THERAPEUTIC CONTINUATION: every |__|__| days or

every |__|__| months □ just when needed

1. On average, how often do you require instrumental examinations ((eg, laboratory tests) for the three different types of patients with schizophrenia?

…NEWLY DIAGNOSED PATIENTS: every |__|__| days or every |__|__| months

□ never

…PATIENTS IN THERAPEUTIC CHANGE: every |__|__| days or every |__|__| months

□ never

…PATIENTS IN THERAPEUTIC CONTINUATION: every |__|__| days or

every |__|__| months □ never

1. In detail, what tests do you request for the three different types of patients? (multiple answers may be possible)

|  | Patients diagnosed for the first time | Patients in who undergo therapeutic change | Patients in therapeutic continuation |
| --- | --- | --- | --- |
| Electrocardiogram |  |  |  |
| Cholesterol blood level |  |  |  |
| Fasting blood sugar level |  |  |  |
| Blood pressure |  |  |  |
| Triglycerides blood levels |  |  |  |
| Serum electrolyte levels |  |  |  |
| Prolactin levels |  |  |  |
| Thyroid hormone levels |  |  |  |
| Sexual hormones (female/male gender) |  |  |  |
| Blood tests of renal function |  |  |  |
| Liver enzymes levels |  |  |  |
| Mammography |  |  |  |
| PAP test |  |  |  |
| Prostate specific antigen test |  |  |  |
| Faecal occult blood test |  |  |  |
| HIV-hepatitis testing |  |  |  |
| Psychometric tests for sexual functioning |  |  |  |
| Drug metabolites |  |  |  |
| Others *(specify) ______________* |  |  |  |

1. Regardless of the type of patient, how often do you investigate the following aspects?

|  | NEVER | ONLY AT THE FIRST VISIT | ONLY FOR PATIENTS IN THERAPEUTC CHANGE | SOMETIMES | OFTEN | ALWAYS, AT EVERY VISIT |
| --- | --- | --- | --- | --- | --- | --- |
| Body weight |  |  |  |  |  |  |
| Waist circumference |  |  |  |  |  |  |
| Sexual dysfunction |  |  |  |  |  |  |
| Tremor |  |  |  |  |  |  |
| Sleep disturbance |  |  |  |  |  |  |
| Gynecomastia |  |  |  |  |  |  |
| Respiratory tract diseases |  |  |  |  |  |  |
| Urinary dysfunctions |  |  |  |  |  |  |
| Musculoskeletal diseases |  |  |  |  |  |  |
| Family history of cardiovascular disease |  |  |  |  |  |  |

1. How often do you investigate the following aspects of everyday life?

|  | NEVER | ONLY AT THE FIRST VISIT | ONLY FOR PATIENTS IN THERAPEUTC CHANGE | SOMETIMES | OFTEN | ALWAYS, AT EVERY VISIT |
| --- | --- | --- | --- | --- | --- | --- |
| Dietary habits |  |  |  |  |  |  |
| Physical activity |  |  |  |  |  |  |
| Substance abuse/dependence  (excluding alcohol) |  |  |  |  |  |  |
| Behavioural addiction (sex, internet gaming, others) |  |  |  |  |  |  |
| Smoking habits |  |  |  |  |  |  |
| Alcohol abuse/use |  |  |  |  |  |  |
| Psychotropic prescribed medications  use/misuse/abuse/dependence |  |  |  |  |  |  |
| Couple relationship |  |  |  |  |  |  |
| Frequency of sexual intercourse or masturbation |  |  |  |  |  |  |
| Sexual orientation |  |  |  |  |  |  |
| Others *(specify) ______________* |  |  |  |  |  |  |

1. In approaching and managing medical comorbidities, do you usually consult a specialist?

|  | NEVER | SOMETIMES | FREQUENTLY |
| --- | --- | --- | --- |
|  |  |  |  |

If the answer is affirmative (ie, if the answer is “sometimes” or “frequently”), which specialist do you consult? (*specify*)________________________________

**DEMOGRAPHICS**

Gender

1 ❑ male

2 ❑ female

Age: |__|__| years

Years of activity

|__|__| years

City of provenance

_____________________

Province of provenance

_____________________

Region of Italy:

| 1 ❑ Piedmont | 11 ❑ Lazio |
| --- | --- |
| 2 ❑ Aosta Valley | 12 ❑ Umbria |
| 3 ❑ Lombardy | 13 ❑ Abruzzo |
| 4 ❑ Liguria | 14 ❑ Molise |
| 5 ❑ Friuli-Venezia Giulia | 15 ❑ Campania |
| 6 ❑ Trentino-Alto Adige | 16 ❑ Puglia |
| 7 ❑ Veneto | 17 ❑ Basilicata |
| 8 ❑ Emilia-Romagna | 18 ❑ Calabria |
| 9 ❑ Tuscany | 19 ❑ Sicily |
| 10 ❑ Marches | 20 ❑ Sardinia |

Name: _____________________________________

Surname: __________________________________

Position in the Register of Medical Practitioners: ________________________

E-mail: ______________________________
